# Synthesis and Characterization of *N*-(Arylcarbamothioyl)-cyclohexanecarboxamide Derivatives: The Crystal Structure of *N*-(Naphthalen-1-ylcarbamothioyl)cyclohexanecarboxamide

**DOI:** 10.3390/molecules14020655

**Published:** 2009-02-10

**Authors:** Cemal Koray Özer, Hakan Arslan, Don VanDerveer, Nevzat Külcü

**Affiliations:** 1 Department of Chemistry, Faculty of Pharmacy, Mersin University, Mersin, TR 33169, Turkey;; 2 Department of Natural Sciences, Fayetteville State University, Fayetteville, NC 28301, USA; 3 Department of Chemistry, Clemson University, Clemson, SC 29634, USA; E-mail: dvander@clemson.edu (D. V.); 4 Department of Chemistry, Faculty of Arts and Science, Mersin University, Mersin, TR 33343, Turkey; E-mail: nkulcu@mersin.edu.tr (N. K.)

**Keywords:** Synthesis, Cyclohexane, Thiourea, Single crystal structure, Pseudo-six-membered ring

## Abstract

A number of *N*-(arylcarbamothioyl)cyclohexanecarboxamide derivatives (aryl substituents: phenyl, 2-chlorophenyl, 3-chlorophenyl, 4-chlorophenyl, *o*-tolyl, *p*-tolyl, 3-methoxyphenyl, 4-methoxyphenyl and naphthalen-1yl) have been synthesized. The compounds obtained were characterized by elemental analyses, IR spectroscopy and ^1^H-NMR spectroscopy. *N*-(naphthalen-1-ylcarbamothioyl)cyclohexanecarboxamide, **H_2_L^9^**, was also characterized by a single crystal X-ray diffraction study. This compound, C_18_H_20_N_2_OS, crystallizes in the triclinic space group *P*ī, with *Z* = 2, and unit cell parameters *a* = 6.9921(14) Å, *b* = 11.002(2) Å, *c* = 12.381(3) Å, *α* = 113.28(3)°, *β* = 99.38(3)°, and *γ* = 101.85(3)°. The cyclohexane ring adopts a chair conformation. The molecular conformation of the compound is stabilized by an intramolecular (N2-H2•••O1) hydrogen bond which forms a pseudo-six-membered ring.

## Introduction

Chelating extractants have been found to be more selective for separating metal ions than solvating reagents and anion exchangers. Some of the most important chelating extractants are thiourea derivatives, which have been known for a long time and are easily synthesized in good yields. Metal complexes of thiourea derivatives have been the subject of considerable study [1,2,3,4,5,6,7,8,9,10,11,12,13]. The presence of hard O- and N- and soft S-donor atoms in the backbones of these ligands enable them to react readily with both transition group and main group metal ions, yielding stable metal complexes, some of which have been shown to exhibit interesting physico-chemical properties and significant biological activities [13,14,15]. They have received particular attention because of their potential use as highly selective reagents for the pre-concentration and separation of metals [16,17,18,19,20]. Some thiourea derivatives have been found to be useful as insecticides, fungicides, herbicides, and plant-growth regulators [13,14,21,22]. In recent years, thiourea derivatives have been the subject of interest because it has been shown that they have antitumor and antifungal bioactivities and inhibitory activities against viruses [15,23,24].

Over the past couple of years we have been focusing on the preparation and characterization of new carboxamides which are bonded with a thiourea group [1,2,3,4,5,25,26,27,28,29,30,31,32]. Taking into consideration all the features described above, we focused our efforts in the present work on the synthesis of carboxamide derivatives. We have now obtained nine novel carboxamide derivatives and, in this paper, we report the preparation and characterization of these new derivatives: *N*-(phenylcarbamothioyl) cyclohexanecarboxamide (**H_2_L^1^**), *N*-(2-chlorophenylcarbamothioyl)cyclohexanecarboxamide (**H_2_L^2^**), *N*-(3-chlorophenylcarbamothioyl)cyclohexanecarboxamide (**H_2_L^3^**), *N*-(4-chlorophenylcarbamothioyl) cyclohexanecarboxamide (**H_2_L^4^**), *N*-(*o*-tolylcarbamothioyl)cyclohexanecarboxamide (**H_2_L^5^**), *N*-(*p*-tolylcarbamothioyl)cyclohexanecarboxamide (**H_2_L^6^**), *N*-(3-methoxyphenylcarbamothioyl)cyclohexane carboxamide (**H_2_L^7^**), *N*-(4-methoxyphenylcarbamothioyl)cyclohexanecarboxamide (**H_2_L^8^**) and *N*-(naphthalen-1-ylcarbamothioyl)cyclohexanecarboxamide (**H_2_L^9^**). The crystal structure of the last compound is also described.

## Results and Discussion

**Figure 1:**
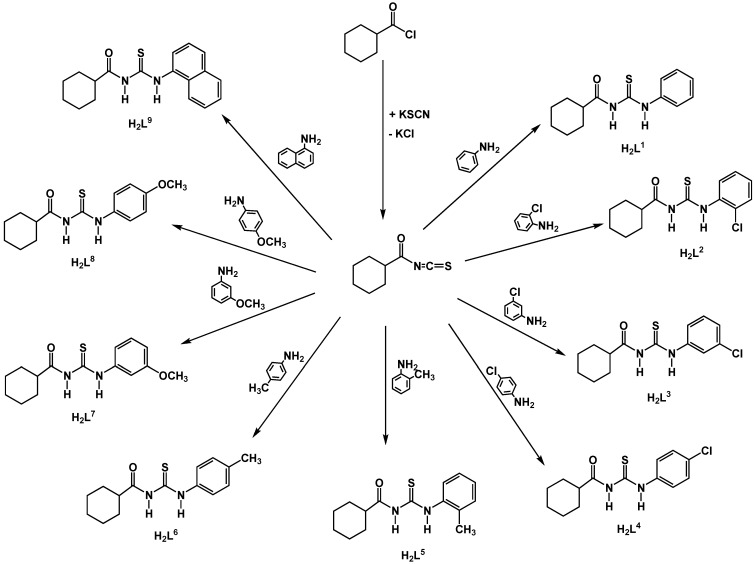
Syntheses of the compounds.

The synthesis of intermediate and target compounds were performed as illustrated in Figure 1. The derivatives were synthesized in excellent yields following the method described by Douglass and Dains [33], which involved the reaction of a cyclohexanecarbonyl chloride with potassium thiocyanate in acetone followed by condensation of the resulting cyclohexanecarbonyl isothiocyanate with the appropriate primary amine. All spectroscopic methods and elemental analyses confirm the proposed structures of the new compounds.

The characteristic IR bands of the synthesized compounds are presented in the Experimental section. The compounds showed two peaks in the 3256-3227 cm^-1^ and 3215-3134 cm^-1^ regions due to the N-H stretching vibrations. This difference between the ν_NH_ stretching vibration frequencies is due to an intramolecular hydrogen bond (X-ray single crystal diffraction data), whereby the carbonyl group is connected to the imine group. Compounds showed a single peak in the 1690-1682 cm^-1^ region, which was due to the C=O stretching vibration. A strong band in the 1252-1238 cm^-1^ region is assigned as the thiocarbonyl group stretching vibration band. These results agree with the literature data [1,2,3,4,5,25,26,33,34,35].

The ^1^H-NMR data of the compounds are presented in the Experimental section. The ^1^H-NMR spectra of the compounds are consistent with the structural results. We discuss only the N-H signal of the investigated compounds. The two N-H signals for the compounds are observed in the 12.15-12.62 ppm region and in the 8.76-9.21 ppm range. Because of the formation of an intramolecular hydrogen bond, the imine group proton participating in the hydrogen bond appears at low field [34]. The other imine proton appears at high field. This data agrees with the results of Li *et al*. and Mansuroglu *et al*. [26,35]. All of these data agree with FT-IR ATR and X-ray single crystal diffraction results.

**Figure 2:**
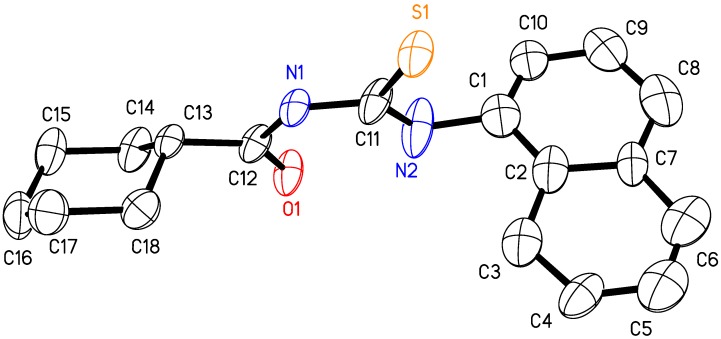
Molecular structure of **H_2_L^9^**. Thermal ellipsoids are shown at the 50% probability level.

The structure of *N*-(naphthalen-1-ylcarbamothioyl)cyclohexanecarboxamide was confirmed by the result of a single crystal X-ray structure determination. Experimental details for data collection and structure refinement are summarized in Table 1. An ORTEP diagram of the molecular structure of **H_2_L^9^** in the crystal form with the corresponding atom numbering scheme is shown in Figure 2. Selected bond lengths and angles can be found in Table 2. The bond lengths of the carbonyl (C12-O1 = 1.218(5) Å) and thiocarbonyl (C11-S1 = 1.670(4) Å) groups of the *N*-(naphthalen-1-ylcarbamothioyl)-cyclohexane carboxamide have typical double-bond character [36,37,38,39,40]. However, the C-N bond lengths for the investigated compound are all shorter than the average single C-N bond length of 1.48 Å, being C12-N1 = 1.388(5) Å, C11-N1= 1.378(5) Å, C11-N2 = 1.340(6) Å, thus showing varying degrees of double bond character [22,39,40,41,42,43,44]. These results are in agreement with the expected delocalization in *N*-(naphthalen-1-ylcarbamothioyl)cyclohexanecarboxamide and confirmed by C11-N1-C12 = 129.7(4)^o^ and C11-N2-C1 = 122.1(4)^o^ angles showing an *sp*^2^ hybridization on the N1 and N2 atoms. As presented in Figure 2, the molecule maintains its *cis-trans* configuration with respect to the position of the naphthalene and cyclohexane groups relative to the thiocarbonyl sulfur atom across the N1-C11 and N2-C11 bonds, respectively [42,43,44]. 

**Table 1:** Summary of crystallographic data and parameters of *N*-(naphthalen-1-ylcarbamothioyl)cyclohexanecarboxamide.

Empirical formula	C_18_H_20_N_2_OS
Formula weight	312.42
Temperature (K)	153(2)
Wavelength (Å)	0.71073
Crystal system	Triclinic
Space group	*P*ī
Unit cell dimensions	
*a* (Å)	6.9921(14)
*b* (Å)	11.002(2)
*c* (Å)	12.381(3)
*α* (°)	113.28(3)
*β* (°)	99.38(3)
*γ* (°)	101.85(3)
*V* (Å3)	824.1(3)
Z	2
*D*_c _(Mg/m^3^)	1.259
Absorption coefficient (mm^-1^)	0.200
F(000)	332
Crystal size (mm^3^)	0.29 x 0.24 x 0.14
*θ* range for data collection (°)	3.37 to 25.05
Index ranges	-8 ≤ *h* ≤ 8
	-10 ≤ *k* ≤ 13
	-14 ≤ *l* ≤ 14
Reflections collected	5439
Independent reflections (*R*_int_)	2841 (0.0261)
Absorption correction	Semi-empirical from equivalents
Refinement method	Full-matrix least-squares on F2
Data / parameters	2841 / 199
Goodness-of-fit on F2	1.104
Final R indices [I>2σ(I)]	R1 = 0.0884, wR2 = 0.2414
R indices (all data)	R1 = 0.1236, wR2 = 0.2990
Largest diff. peak and hole (e.Å-3)	1.208 and -0.578

**Table 2:** Selected bond lengths (Å) and angles (^o^).

**Bond lengths**				
O1-C12	1.218(5)		N2-C11	1.340(6)
S1-C11	1.670(4)		N2-C1	1.466(6)
N1-C12	1.388(5)		C12-C13	1.504(6)
N1-C11	1.378(5)			
				
**Bond angles**				
C12-N1-C11	129.7(4)		N1-C11-S1	119.8(3)
C11-N2-C1	122.1(4)		O1-C12-N1	121.7(4)
N1-C11-N2	116.3(4)		O1-C12-C13	124.2(4)
N2-C11-S1	123.8(3)		N1-C12-C13	114.0(3)

**Figure 3:**
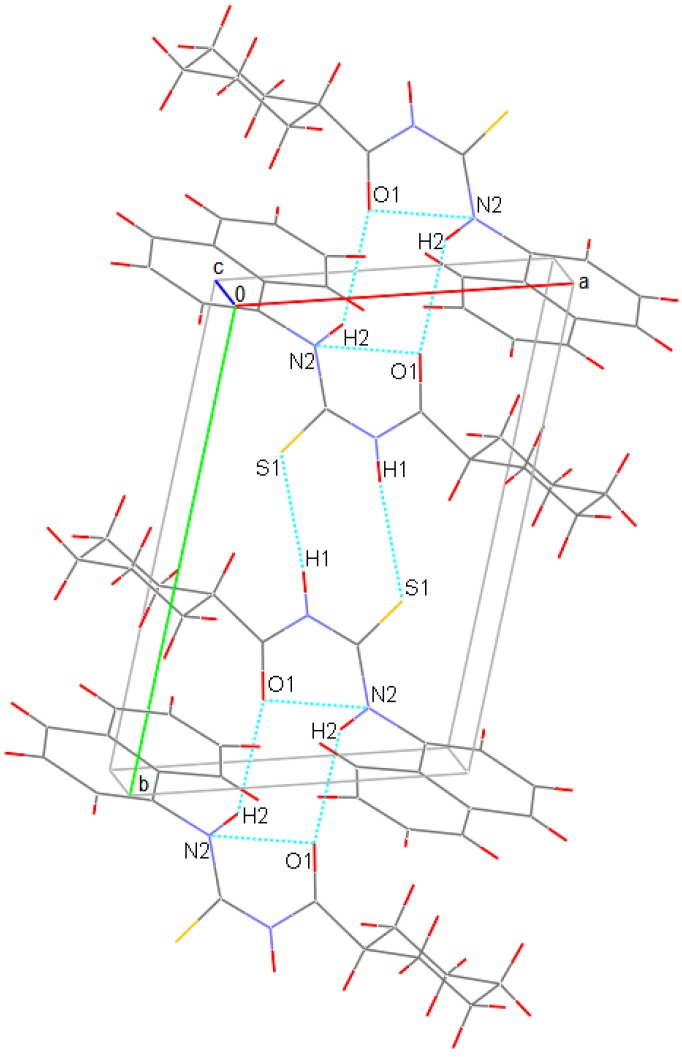
Packing diagram for **H_2_L^9^** with hydrogen bonds as dotted lines [46].

The conformation of the **H_2_L^9^** molecule with respect to the thiocarbonyl and carbonyl moieties is twisted, as reflected by the torsion angles O1-C12-N1-C11, C12-N1-C11-N2, and S1-C11-N1-C12 of -1.23^o^, 6.42^o^ and 176.83^o^, respectively. The O1-C12-N1-C11-N2 plane has a maximum deviation of 0.038(4) Å for C11 atom. However, the central carbonyl thiourea moiety (S1-C11-N2-N1-C12-O1) connecting the naphthalene and cyclohexane groups is almost planar, with the O1 atom deviating by 0.047(4) Å. In addition, the naphthalene rings are essentially planar. The cyclohexane ring exhibits a puckered conformation, with puckering parameters [45], *q*_2_ = 0.014(6) Å, *q*_3_ = -0.572(6) Å, *Q*_T_ = 0.571(6) Å, *θ* = 180.0(6)^o^ and *ϕ* = 192(23)^o^. The largest deviations from the mean plane are 0.241(5) Å for atom C13 and 0.225(6) Å for atom C16. This ring puckering analysis shows that the cyclohexane ring has a chair conformation, with equatorial substitution at C13 for C12. In the crystal structure, the molecules are packed as dimers, via intermolecular contacts N1-H1•••S1*^i^*, with N-H 0.91 Å, H-S 2.48 Å, N-H•••S 172^o^ and N2-H2•••O1*^ii^*, with N-H 0.91 Å, H-O 2.48 Å, N-H•••O 139^o^ symmetry code: (*i*) -3-*x*, 1-*y*, -1-*z*; (*ii*) -3-*x*, -*y*, -1-*z* as shown in Figure 3. The intramolecular hydrogen bonding N2-H2•••O1 maintains the six-membered ring formation of the O1-C12-N1-C11-N2 plane; N2-H2•••O1 with N-H 0.91 Å, H•••O 1.98 Å, N-H•••O 132^o^ (Figure 3). The bond distances and angles observed in the H_2_L^9^ molecule is consistent with those reported for the other similar thiourea derivative [25]. It appears that this intramolecular hydrogen bonding is also present in the solution state. We base this conclusion on both the IR and NMR evidence cited above and the coordination behavior of the investigated compound. These thiourea derivatives have one imine group like *N*,*N*-dialkyl-*N*’-benzoylthioureas [1,2,3,4,5]. These compounds easily coordinate to a metal atom via both sulfur and oxygen atoms. However, for the title type thiourea compounds [R(Ar)-CO-NH-CS-NH-R(Ar)] we observe that the carbonyl oxygen atom of the thiourea derivatives does not participate in the coordination with transition metal atoms because of the strong N-H•••O intramolecular hydrogen bond [26].

## Experimental

### General

All chemicals used for the preparation of the compounds were of reagent grade quality. The room temperature attenuated total reflection Fourier transform infrared (FT-IR ATR) spectra of the all synthesized compounds were registered using a Varian FTS1000 FT-IR spectrometer with a Diamond/ZnSe prism (4000–525 cm^−1^; number of scans: 250; resolution: 1 cm^−1^). All ^1^H-NMR spectra were recorded on a Bruker DPX-400 spectrometer, using CDCl_3_ as the solvent and TMS as an internal standard. C, H and N analyses were carried out on a Carlo Erba MOD 1106 elemental analyzer. Single crystal X-ray data were collected on a Rigaku Mercury AFC8S system with a Mercury CCD detector using graphite-monochromated MoK_α_ radiation (λ = 0.71073 Å). The structure was solved by direct methods and refined by using full-matrix least-squares techniques (on *F*^2^) [47]. Data were corrected for Lorentz and polarization effects and for absorption. The latter correction was made using REQABA, a multi-scan technique [48]. All non-hydrogen atoms were refined anisotropically. Further details concerning data collection and refinement are given in Table 1.

### Synthesis of the compounds

The compounds were prepared by a procedure similar to that reported in the literature [1,2,3,4,5,22]. A solution of cyclohexanecarbonyl chloride (0.005 mole) in acetone (50 mL) was added dropwise to a suspension of potassium thiocyanate (0.005 mole) in acetone (50 mL). The reaction mixture was heated under reflux for 30 min, and then cooled to room temperature. A solution of the appropriate primary amine (0.005 mole) (2-chlorobenzenamine, 3-chlorobenzenamine, 4-chlorobenzenamine, *o*-toluidine, *p*-toluidine, 3-methoxybenzenamine, 4-methoxybenzenamine, and naphthalen-1-amine) in acetone (30 mL) was added to the mixture for 15 min at room temperature and stirred for 2 h. Hydrochloric acid (0.1 N, 300 mL) was added and the solution was filtered. The solid product was washed with water and purified by recrystallization from an ethanol-dichloromethane mixture (1:2).

*N-(phenylcarbamothioyl)cyclohexanecarboxamide* (**H_2_L^1^**): White. Yield: 91 %. M.p.: 162-164 ^o^C. Anal. calcd. for C_14_H_18_N_2_OS: C 64.1; H 6.9; N 10.7. Found: C 64.3; H 7.0; N 10.6 %. FT-IR (cm^-1^): ν(NH) 3244, 3151 (m), ν(Ar-CH) 3065, 3034 (w), ν(CH) 2933, 2859 (s), ν(C=O) 1686 (s), ν(C=S) 1246 (s). ^1^H-NMR: δ 12.48 (s, 1H, CSN*H*), 8.97 (s, 1H, CON*H*), 7.67 (d, 2H, Ar-*H*), 7.42 (t, 2H, Ar-*H*), 7.28 (t, 1H, Ar-*H*), 2.30 (tt, *J =* 10.1, 3.2, 1H, C*H*, cyclohexane:Ch), 1.99 (d, *J =* 2.1, 1H, C*H*, Ch), 1.94 (d, *J =* 2.0, 1H, C*H*, Ch), 1.88 (m, 1H, C*H*, Ch), 1.84 (t, *J =* 1.9, 1H, C*H*, Ch), 1.73 (m, 2H, C*H*, Ch), 1.52 (m, 2H, C*H*, Ch), 1.31 (m, 2H, C*H*, Ch).

*N-(2-chlorophenylcarbamothioyl)cyclohexanecarboxamide* (**H_2_L^2^**): White. Yield: 93 %. M.p.: 136-138 ^o^C. Anal. calcd. for C_14_H_17_N_2_OSCI: C 56.7; H 5.8; N 9.4. Found: C 55.9; H 5.7; N 9.6 %. FT-IR (cm^-1^): ν(NH) 3227, 3197 (m), ν(Ar-CH) 3097, 3035, 2995 (vw), ν(CH) 2937, 2926, 2854 (m), ν(C=O) 1686 (s), ν(C=S) 1246 (s). ^1^H-NMR: δ 12.60 (s, 1H, CSN*H*), 8.76 (s, 1H, CON*H*), 7.49 (d, 1H, Ar-*H*), 7.46 (d, 1H, Ar-*H*), 7.34 (td, 1H, Ar-*H*), 7.23 (td, 1H, Ar-*H*), 2.29 (tt, *J =* 10.3, 3.6, 1H, C*H*, Ch), 2.01 (d, *J =* 2.3, 1H, C*H*, Ch), 1.97 (d, *J =* 2.2, 1H, C*H*, Ch), 1.89 (t, *J =* 2.1, 1H, C*H*, Ch), 1.85 (m, 1H, C*H*, Ch), 1.73 (m, 2H, C*H*, Ch), 1.55 (m, 2H, C*H*, Ch), 1.31 (m, 2H, C*H*, Ch).

*N-(3-chlorophenylcarbamothioyl)cyclohexanecarboxamide* (**H_2_L^3^**): White. Yield: 89 %. M.p.: 163-165 ^o^C. Anal. calcd. for C_14_H_17_N_2_OSCI: C 56.7; H 5.8; N 9.4. Found: C 57.6; H 5.8; N 9.5 %. FT-IR (cm^−1^): ν(NH) 3242, 3134 (m), ν(Ar-CH) 3064, 3026 (vw), ν(CH) 2937, 2929, 2852 (m), ν(C=O) 1688 (vs), ν(C=S) 1240 (s). ^1^H-NMR: δ 12.56 (s, 1H, CSN*H*), 9.07 (s, 1H, CON*H*), 7.82 (t, 1H, Ar-*H*), 7.53 (dt, 1H, Ar-*H*), 7.34 (t, 1H, Ar-*H*), 7.25 (dt, 1H, Ar-*H*), 2.30 (tt, *J =* 10.7, 3.8, 1H, C*H*, Ch), 1.97 (d, *J =* 2.4, 1H, C*H*, Ch), 1.93 (d, *J =* 2.2, 1H, C*H*, Ch), 1.87 (t, *J =* 2.2, 1H, C*H*, Ch), 1.83 (m, 1H, C*H*, Ch), 1.74 (m, 2H, C*H*, Ch), 1.51 (m, 2H, C*H*, Ch), 1.31 (m, 2H, C*H*, Ch).

*N-(4-chlorophenylcarbamothioyl)cyclohexanecarboxamide* (**H_2_L^4^**): White. Yield: 86 %. M.p.: 184-186 ^o^C. Anal. calcd. for C_14_H_17_N_2_OSCI: C 56.7; H 5.8; N 9.4. Found: C 56.6; H 5.9; N 9.6 %. FT-IR (cm^−1^): ν(NH) 3254, 3153 (m), ν(Ar-CH) 3066, 3034 (w), ν(CH) 2941, 2924, 2858 (m), ν(C=O) 1686 (vs), ν(C=S) 1252 (s). ^1^H-NMR: δ 12.51 (s, 1H, CSN*H*), 9.01 (s, 1H, CON*H*), 7.63 (d, 2H, Ar-*H*), 7.38 (d, 2H, Ar-*H*), 2.29 (tt, *J =* 10.4, 3.5, 1H, C*H*, Ch), 1.97 (s, 1H, C*H*, Ch), 1.93 (s, 1H, C*H*, Ch), 1.87 (d, *J =* 2.1, 1H, C*H*, Ch), 1.84 (d, *J =* 2.3*,* 1H, C*H*, Ch), 1.75 (m, 2H, C*H*, Ch), 1.51 (m, 2H, C*H*, Ch), 1.30 (m, 2H, C*H*, Ch).

*N-(o-tolylcarbamothioyl)cyclohexanecarboxamide* (**H_2_L^5^**): White. Yield: 91 %. M.p.: 148-150 ^o^C. Anal. calcd. for C_15_H_20_N_2_OS: C 65.2; H 7.3; N 10.1. Found: C 65.8; H 7.4; N 10.3 %. FT-IR (cm^-1^): ν(NH) 3236, 3165 (m), ν(Ar-CH) 3065, 3028 (w), ν(CH) 2932, 2853 (m), ν(C=O) 1686 (s), ν(C=S) 1246 (m). ^1^H-NMR: δ 12.15 (s, 1H, CSN*H*), 9.16 (s, 1H, CON*H*), 7.72 (dd, 1H, Ar-*H*), 7.31-7.22 (m, 3H, Ar-*H*), 2.34 (s, 3H, Ar-C*H*_3_), 2.32 (tt, *J =* 10.3, 3.3, 1H, C*H*, Ch), 2.00 (d, *J =* 2.4, 1H, C*H*, Ch), 1.95 (s, 1H, C*H*, Ch), 1.87 (m, 1H, C*H*, Ch), 1.83 (m, 1H, C*H*, Ch), 1.74 (m, 2H, C*H*, Ch), 1.52 (m, 2H, C*H*, Ch), 1.30 (m, 2H, C*H*, Ch).

*N-(p-tolylcarbamothioyl)cyclohexanecarboxamide* (**H_2_L^6^**): White. Yield: 90 %. M.p.: 176-178 ^o^C. Anal. calcd. for C_15_H_20_N_2_OS: C 65.2; H 7.3; N 10.1. Found: C 66.0; H 7.3; N 10.3 %. FT-IR (cm^-1^): ν(NH) 3240, 3172 (m), ν(Ar-CH) 3034 (w), ν(CH) 2932, 2859 (m), ν(C=O) 1686 (s), ν(C=S) 1250 (s). ^1^H-NMR: δ 12.38 (s, 1H, CSN*H*), 8.98 (s, 1H, CON*H*), 7.52 (d, 2H, Ar-*H*), 7.22 (d, 2H, Ar-*H*), 2.38 (s, 3H, Ar-C*H*_3_), 2.29 (tt, *J =* 10.2, 3.7, 1H, C*H*, Ch), 1.98 (s, 1H, C*H*, Ch), 1.94 (s, 1H, C*H*, Ch), 1.87 (m, 1H, C*H*, Ch), 1.83 (m, 1H, C*H*, Ch), 1.74 (m, 2H, C*H*, Ch), 1.51 (m, 2H, C*H*, Ch), 1.31 (m, 2H, C*H*, Ch).

*N-(3-methoxyphenylcarbamothioyl)cyclohexanecarboxamide* (**H_2_L^7^**): White. Yield: 93 %. M.p.: 94-96 ^o^C. Anal. calcd. for C_15_H_20_N_2_O_2_S: C 61.6; H 6.9; N 9.6. Found: C 62.5; H 6.8; N 9.6 %. FT-IR (cm^-1^): ν(NH) 3256, 3215 (m), ν(Ar-CH) 3034, 3005 (w), ν(CH) 2943, 2939, 2916, 2862, 2849, 2841 (m), ν(C=O) 1690 (s), ν(C=S) 1246 (s). ^1^H-NMR: δ 12.53 (s, 1H, CSN*H*), 9.08 (s, 1H, CON*H*), 7.44 (t, 1H, Ar-*H*), 7.30 (d, 1H, Ar-*H*), 7.18 (dd, 1H, Ar-*H*), 6.83 (dd, 1H, Ar-*H*), 3.83 (s, 3H, OC*H*_3_), 2.30 (tt, *J =* 10.3, 3.2, 1H, C*H*, Ch), 1.98 (d, *J =* 2.5, 1H, C*H*, Ch), 1.93 (d, *J =* 2.4, 1H, C*H*, Ch), 1.86 (m, 1H, C*H*, Ch), 1.83 (m, 1H, C*H*, Ch), 1.73 (m, 2H, C*H*, Ch), 1.51 (m, 2H, C*H*, Ch), 1.30 (m, 2H, C*H*, Ch).

*N-(4-methoxyphenylcarbamothioyl)cyclohexanecarboxamide* (**H_2_L^8^**): White. Yield: 94 %. M.p.: 167-169 ^o^C. Anal. calcd. for C_15_H_20_N_2_O_2_S: C 61.6; H 6.9; N 9.6. Found: C 61.3; H 6.8; N 9.4 %. FT-IR (cm^-1^): ν(NH) 3236, 3202 (m), ν(Ar-CH) 3041, 3013 (w), ν(CH) 2934, 2928, 2850, 2843 (m), ν(C=O) 1686 (s), ν(C=S) 1238 (s). ^1^H-NMR: δ 12.29 (s, 1H, CSN*H*), 8.93 (s, 1H, CON*H*), 7.53 (dt, 2H, Ar-*H*), 6.94 (dt, 2H, Ar-*H*), 3.84 (s, 3H, OC*H*_3_), 2.28 (tt, *J =* 10.4, 3.4, 1H, C*H*, Ch), 1.98 (d, *J =* 2.4, 1H, C*H*, Ch), 1.93 (d, *J =* 2.4, 1H, C*H*, Ch), 1.87 (m, 1H, C*H*, Ch), 1.83 (m, 1H, C*H*, Ch), 1.74 (m, 2H, C*H*, Ch), 1.51 (m, 2H, C*H*, Ch), 1.30 (m, 2H, C*H*, Ch).

*N-(naphthalen-1-ylcarbamothioyl)cyclohexanecarboxamide* (**H_2_L^9^**): White. Yield: 92 %. M.p.: 176-178 ^o^C. Anal. calcd. for C_18_H_20_N_2_OS: C 69.2; H 6.5; N 9.0. Found: C 69.9; H 6.4; N 8.9 %. FT-IR (cm^-1^): ν(NH) 3233, 3169 (m), ν(Ar-CH) 3051, 3030 (w), ν(CH) 2932, 2922, 2852 (m), ν(C=O) 1682 (s), ν(C=S) 1242 (m). ^1^H-NMR: δ 12.62 (s, 1H, CSN*H*), 9.21 (s, 1H, CON*H*), 7.98-7.81 (m, 4H, Ar-*H*), 7.58-7.48 (m, 3H, Ar-*H*), 2.30 (tt, *J =* 10.2, 3.3, 1H, C*H*, Ch), 1.98 (s, 1H, C*H*, Ch), 1.94 (s, 1H, C*H*, Ch), 1.83 (d, *J =* 2.3, 1H, C*H*, Ch), 1.79 (d, *J =* 2.2, 1H, C*H*, Ch), 1.69 (m, 2H, C*H*, Ch), 1.52 (m, 2H, C*H*, Ch), 1.25 (m, 2H, C*H*, Ch).

## Supplementary material

Crystallographic data for the structure reported in this paper have been deposited at the Cambridge Crystallographic Data Centre (CCDC) with quotation number CCDC-675756 for **H_2_L^9^** and can be obtained free of charge on application to CCDC 12 Union Road, Cambridge CB2 1EZ, UK Fax: (internat.) + 44(1223)336-033, E-mail: deposit@ccdc.cam.ac.uk.
